# Knowledge, Attitude, and Practice Toward Dietary Salt Intake Among Medical Students at King Abdulaziz University

**DOI:** 10.7759/cureus.51911

**Published:** 2024-01-08

**Authors:** Areej Algarni, Reema A Ayoub, Zahra A Alkhadrawi, Tharwa O Barnawi, Israa A Bajafar, Tahani Y Samkari, Rana Alkuhili, Khaled A Yaghmour

**Affiliations:** 1 College of Medicine, Faculty of Medicine, King Abdulaziz University, Jeddah, SAU; 2 Family Medicine Department, Faculty of Medicine, King Abdulaziz University, Jeddah, SAU

**Keywords:** knowledge attitude and practice, salt use, knowledge attitude practices studies, cardio, idiopathic hypertension, cardio vascular disease risk factors, dietary practices, dietary sodium

## Abstract

Background

Since the beginning of the century, dietary patterns have been changing rapidly due to evolving lifestyles, restaurants that cater to dietary restrictions, etc. As a result, populations started consuming a large amount of salt in their diets. Years of research have found that high salt intake is strongly related to many serious health problems like hypertension and cardiovascular diseases. This study's objective is to evaluate knowledge, attitudes, and practices regarding dietary salt intake among medical students at King Abdulaziz University (KAU) in Jeddah, Saudi Arabia, and to identify barriers and concerns related to optimum dietary sodium intake.

Method

A cross-sectional study done at KAU among 310 students using an online questionnaire included items to assess their knowledge, attitudes, and practices related to dietary salt intake. There were 24 questions to assess knowledge, six questions to assess attitude, and eight questions to assess practice.

Results

The mean age of the participants was 21.52 ± 1.94 years; 180 of the 57.5% were female. A score of “1” was given to the right answer for knowledge, positive attitude, and correct practice. The mean knowledge, attitudes, and practices scores were 16.99 ± 3.8, 3.03 ± 1.46, and 2.13 ± 1.34, respectively. The percentage of poor, fair, and good knowledge levels regarding dietary salt intake among studied students was 72:23%, 210:67.1%, and 31:9.9%. While the prevalence of negative, fair, and positive attitudes was 111:35.5%, 141:45%, and 161:9.5%. As for practice level, none of the students had good practice, while 263:84% and 50:16% had poor and fair practice, respectively.

Conclusion

In conclusion, the majority of students were aware that a high-salt diet can result in serious health issues. They were also uncertain of whether their salt intake was extremely high or not, which is consistent with the fact that they were ignorant of the daily salt intake guidelines. The findings in our study can stand as a reference point for salt-related knowledge, attitude, and practice (KAP) studies to help further future research in Saudi Arabia and other countries. Multi-sector coordination between the food suppliers, health agencies, and government is necessary to increase public awareness, decrease the salt content of food, and lower individual salt consumption in Saudi Arabia.

## Introduction

Since the beginning of the century, dietary patterns have been changing rapidly due to evolving lifestyles, restaurants that cater to dietary restrictions, accessibility to different cuisines, grocery store convenience, and the availability of fast food and junk food [1].

As a result, populations start consuming a large amount of processed food, which plays a major role in the increase of dietary sodium intake [1]. Moreover, the rates of non-communicable diseases (NCDs) are increasing rapidly, and high salt consumption is considered a significant risk factor for many of them, including hypertension and cardiovascular diseases [2]. In addition to high blood pressure and cardiovascular diseases, high salt intake is also found to be associated with osteoporosis, kidney stone formation, obesity, and gastric and nasopharyngeal cancers [3,4]. In fact, according to the WHO, excessive salt consumption is a silent killer that contributed to the deaths of around three million people in 2017 [5].

This seems to be a common problem in Saudi Arabia, where the sodium intake levels were found to be higher than recommended daily rates by the WHO [6]. Additionally, the prevalence of hypertension and diabetes has reached epidemic levels in the country [7].

Due to these statistics, limiting dietary sodium intake has been recommended as an approach to lowering blood pressure, and therefore helping prevent and control NCD. Less than 2300 mg of sodium should be consumed per day, according to the American Heart Association (AHA) [8]. As a result, several countries began strategies to lower dietary sodium intake by combining many procedures, such as educating the public, labeling the food, and collaborating with food sectors to reduce the levels of sodium in processed food [5].

To reduce sodium levels in Saudi Arabia, the reduction of salt in local bakeries that primarily produce bread was implemented as an effective strategy [9]. Evaluating sodium intake knowledge and practices is critical for developing a plan to reduce sodium intake. Currently, there is an insufficient number of salt-related knowledge, attitude, and practice (KAP) studies in Saudi Arabia, particularly in Jeddah. Medical students are a crucial demographic since they represent the future healthcare professionals tasked with providing counseling and guidance and raising awareness among the general community.

Thus, the purpose of this study was to assess medical students' knowledge, attitude, and behavior regarding dietary sodium intake at King Abdulaziz University (KAU) in Jeddah, Saudi Arabia, and to identify barriers and concerns related to the optimum dietary sodium intake.

## Materials and methods

Study design

Cross-sectional research was conducted at King Abdulaziz University, Jeddah, Saudi Arabia, throughout the months of November 2022 and January 2023.

Study participants

The inclusion criteria in this study included all King Abdulaziz University medical students of both genders above 18 years old. The exclusion criteria were all the students under the age of 18 and those who refused to participate in this study. A sample size of 310 students was determined by using the Raosoft web-based sample size calculating software. This was calculated using a margin of error of 5% and a confidence interval of 95%. 

Data collection

A questionnaire was used and distributed online through email, social media platforms, and interviews. The questionnaire begins with consent taken from participants before starting data collection. The first section involved demographic data, including the students’ age, gender, and academic level. The second section included items to evaluate students’ knowledge, attitude, and practice related to dietary salt consumption; these questions were designed according to those used in previous KAP studies related to salt [10-14]. The steps of the KAP survey model were guided by the Food and Agriculture Organization of the United Nations guidelines for assessing nutrition-related knowledge, attitudes, and practices and previous WHO guidance in KAP surveys [15,16].

To assess the knowledge levels, 24 questions were asked, assessing the awareness of the daily salt intake recommendations, the difference between sodium and salt, and the amount of salt that can cause health problems. With each right knowledge, a score of “one” was given. It also included a question assessing awareness regarding five serious health problems associated with high salt intake that had more than one correct answer (a sum score of five), leaving a score ranging from 0 to 29 for knowledge.

To assess the attitude levels, six questions were asked, and for every positive attitude, a score of “one” was given, leaving a score ranging from zero to six for attitude. These questions involved the students’ perceptions toward their amount of daily salt consumption, the importance of reducing salt intake, and the consumption of processed food. To assess the practice levels, eight questions were asked, and for every right practice, a score of “one” was given; thus, a score range of zero to eight was present for the practice. This involved questions about the students’ daily serving of vegetables and fruits, whether they restrict eating outside, check food labels for salt content, or add salt at the table.

If the participants answered less than 50% of the questions accurately, they were considered to have a limited level of knowledge, attitude, or practice. If they answered between 50% and 75% accurately, they were classified as having a fair level of knowledge, attitude, or practice. If they answered more than 75% of the questions accurately, they were considered to have a high level of knowledge, positive attitude, or good practice. Furthermore, barriers related to salt reduction practices were assessed, such as the inaccessibility of low-sodium foods when dining outside, the taste of the low-sodium food, insufficient awareness of which foods to avoid, and the lack of information regarding the risks of consuming a high-sodium diet, which the participants answered with a five-point Likert scale of agreement (strongly agree, agree, neutral, disagree, and strongly disagree). 

Ethical considerations

The study was conducted with the approval of the King Abdulaziz University Research Ethics Committee in Jeddah, Saudi Arabia.

Statistical analysis 

SPSS version 26 (IBM Corp., Armonk, New York, USA) was used to statistically analyze the data. The chi-squared test (χ^2^) was applied to categorical variables (e.g., the students’ beliefs toward daily salt intake recommendations, the importance of reducing salt intake, etc.) that were expressed in numbers and percentages to determine the relationship between the variables. Mean and standard deviation were used to express quantitative data, while Mann-Whitney and Kruskal-Wallis tests were applied to non-parametric variables. Using Spearman's test, correlation analysis was carried out. A p-value of less than 0.05 was regarded as statistically significant.

## Results

The total number of participants in this study was 313 students, with the mean age of the students being 21.52 ± 1.94 years. The majority of the students were females (180 (57.5%)), and the highest percentage in regards to academic years belonged to the fourth academic year (76 (24.3%)), as shown in Table 1.

**Table 1:** Distribution of studied students according to their age, gender, and academic level (n=313).

Variable	No. (%)
Age	21.52 ± 1.94
Gender	
Female	180 (57.5)
Male	133 (42.5)
Academic year	
2nd	59 (18.8)
3rd	63 (20.1)
4th	76 (24.3)
5th	48 (15.3)
6th	67 (21.4)

Regarding the knowledge section, as shown in Table 2, 253 (80.5%) of students thought that there was a daily sodium intake recommendation, and 182 (58.1%) students reported that they understood the distinction between salt and sodium. Furthermore, most of the students (300: 95.8%) reported that consuming excessive amounts of salt may result in significant health problems, and the most commonly mentioned health problems were high blood pressure (no. 266: 84.9%) and kidney stones (no. 195: 62.3%). Of the students, 280 (89.5%) reported that it’s necessary to reduce salt intake. Only 67 of the students (21.4%) knew that the health professionals recommend 5 g (one teaspoon) cutting back on salt intake, and 99 of students (31.6%) knew that processed foods (e.g., bread or cheese) are the main source of salt in the Saudi diet.

**Table 2:** Distribution of studied students according to their responses to knowledge items about dietary salt intake (n=313). “*” Represents the correct answer.

Variable	No. (%)
Do you believe there is a daily sodium intake recommendation?	
I don’t know	43 (13.7)
No	17 (5.4)
Yes	253 (80.5)
Do you understand the diﬀerence between sodium and salt?	
I don’t know	42 (13.4)
No	89 (28.4)
Yes	182 (58.1)
Do you think consuming too much salt could cause serious health problems?	
I don’t know	7 (2.2)
No	6 (1.9)
Yes	300 (95.8)
If you answer yes: what type of health problems?	
High blood pressure	266 (84.9)
Osteoporosis	54 (17.2)
Stomach cancer	32 (10.2)
Kidney stones	195 (62.3)
Stroke	65 (20.7)
All of the above	45 (14.3)
I don’t know	6 (1.9)
Do you think it’s necessary to reduce salt intake?	
I don’t know	21 (6.7)
No	12 (3.8)
Yes	280 (89.5)
Health professionals recommend cutting back on salt intake. What do you believe is the recommended amount?	
10 g (two teaspoons)	11 (3.5)
15 g (three teaspoons)	3 (1)
3 g (1/2 teaspoon)	44 (14.1)
5 g (one teaspoon)*	67 (21.4)
8 g (one and a 1/2 teaspoons)	32 (10.2)
I don’t know	156 (49.8)
What do you believe to be the primary source of salt in the Saudi diet?	
Adding salt when cooking and/or serving	179 (57.2)
Natural food source	14 (4.5)
Processed food (e.g., bread or cheese)*	99 (31.6)
I don't know	21 (6.7)

The participants' distribution about knowledge of sodium content in certain diets is shown in Table 3. The majority of the participants 210 (67.1%) had a fair dietary salt-related knowledge level. The most accurate reported information was 265 (84.7%) “pizza contains high sodium content,” 256 (81.8%) “instant noodles contains high sodium content,” 230 (73.5%) “fruits contain low sodium content,” 228 (72.8%) “manaeesh (traditional thyme or cheese-filled pies) contains high sodium content,” 227 (72.5%) “cheese contains high sodium content,” and 225 (71.9%) “bottled salad dressing contains high sodium content.” While the least accurate reported answers were 70 (22.4%) “corn flakes contain high sodium content” and 72 (23%) “rice contains low sodium content.”

**Table 3:** Distribution of studied students according to their responses to knowledge items related to sodium content in certain diets (n=313). “*” Represents the correct value of salt content in the food (variable).

Variable	High no. (%)	Medium no. (%)	Low no. (%)
Fruits	13 (4.2)	70 (22.4)	230 (73.5)*
Fresh vegetables	15 (4.8)	106 (33.9)	192 (61.3)*
Frozen vegetables	44 (14.1)	129 (41.2)	129 (41.2)*
Canned vegetables	156 (49.8)*	88 (28.1)	69 (22)
Olive oil	35 (11.2)	123 (39.3)	155 (49.5)*
Bread	91 (29.1)	175 (55.9)*	47 (15)
Manaeesh-traditional thyme or cheese-filled pies	228 (72.8)*	73 (23.3)	12 (3.8)
Traditional pies	189 (60.4)*	110 (35.1)	14 (4.5)
Pizza	265 (84.7)*	39 (12.5)	9 (2.9)
Rice	68 (21.7)	173 (55.3)	72 (23)*
Cheese	227 (72.5)*	74 (23.6)	12 (3.8)
Milk	43 (13.7)	151 (48.2)	119 (38)*
Soy sauce	217 (69.3)*	73 (23.3)	23 (7.3)
Ketchup	205 (65.5)*	86 (27.5)	22 (7)
Corn ﬂakes	70 (22.4)*	159 (50.8)	84 (26.8)
Chicken cubes	215 (68.7)*	87 (27.8)	11 (3.5)
Instant noodles	256 (81.8)*	51 (16.3)	6 (1.9)
Bottled salad dressings	225 (71.9)*	74 (23.6)	14 (4.5)

The students’ attitude demonstrated in Table 4 shows that 164 (52.2%) thought that lowering salt intake was essential. Of them, 215 (68.7%) agreed that laws should be passed to restrict the quantity of salt that is added to processed foods, and 205 (65.5%) agreed that lower salt options are hard to find at restaurants or cafes. Of the students, 174 (55.6%) agreed that information about sodium that is displayed on food labels is not always clear, 172 (55%) agreed that decreasing dietary salt will promote health, and 216 (69%) agreed that adding salt to food is necessary to give it flavor.

**Table 4:** Distribution of studied students according to their responses to attitude items towards dietary salt intake (n=313).

Variable	Very important no. (%)	Slightly important no. (%)	Not important no. (%)
How important is it to you to reduce your salt	164 (52.2)	138 (44.1)	11 (3.5)
Please choose the following statements	Agree	Neutral	Disagree
Laws should be passed to restrict the quantity of salt that is added to processed foods	215 (68.7)	74 (23.6)	24 (7.7)
I find that lower salt options are hard to find at restaurants or cafes.	205 (65.5)	86 (27.5)	22 (7)
Information about sodium that is displayed on food labels is not always clear	174 (55.6)	100 (31.9)	39 (12.5)
If I cut back on the salt in my diet, my health would improve	172 (55)	97 (31)	44 (14.1)
I think adding salt to food is necessary to give it ﬂavor	216 (69)	78 (24.9)	19 (6.1)

According to Table 5, showing the students’ practices, it was found that 211 (67.4%) of students thought that they were consuming the proper amount of salt, and 121 (38.7%) were consuming the daily serving of vegetables and fruits. Of them, only 69 (22%) were often trying to restrict eating outside, 58 (18.5%) often lower the amount of salt in food, and only 17 of the students (5.4%) inspect the salt content of food labels. Only 43 (13.7%) avert adding salt during cooking, 47 (15%) often add salt at the table, and 50 (16%) consume the same salt amount as advised per day.

**Table 5:** Distribution of studied students according to their responses to practice items related to dietary salt intake (n=313).

Variable	No. (%)
How much salt do you believe you consume?	
A little amount	37 (11.8)
The right amount	211 (67.4)
Too much salt	65 (20.8)
Do you consume a daily serving of vegetables and fruits?	
I don’t know	30 (9.6)
No	162 (51.8)
Yes	121 (38.7)
Do you try to restrict eating outside?	
Never	28 (8.9)
Often	69 (22)
Rarely	58 (18.5)
Sometimes	158 (50.5)
Do you try to lower the amount of salt in your food?	
Never	65 (20.8)
Often	58 (18.5)
Rarely	73 (23.3)
Sometimes	117 (37.4)
Do you check food labels for salt content?	
Never	142 (54.4)
Often	17 (5.4)
Rarely	106 (33.9)
Sometimes	48 (15.3)
Do you avoid adding salt during cooking	
Never	124 (39.6)
Often	43 (13.7)
Rarely	68 (21.7)
Sometimes	78 (24.9)
Do you add salt at the table?	
Never	100 (31.9)
Often	47 (15)
Rarely	97 (31)
Sometimes	69 (22)
How much salt do you consume per day compared to the recommended amount?	
I consume less than the advised amount	35 (11.2)
I consume more than the advised amount	52 (16.6)
I consume the same amount as advised	50 (16)
I don’t know	176 (56.2)

The most common barrier to reducing salt or sodium in students’ diet was the taste of the low-salt food (171: 54.6%), as shown in Table 6.

**Table 6:** Barriers to reduce salt or sodium in participants' diet.

Variable	Strongly agree no. (%)	Agree no. (%)	Neutral no. (%)	Disagree no. (%)	Strongly disagree no. (%)
Not enough time to check salt levels in food labels	61 (19.5)	83 (26.5)	107 (34.2)	47 (15)	15 (4.8)
Inaccessibility of low-sodium foods when dining outside	129 (41.2)	114 (36.4)	62 (19.8)	4 (1.3)	4 (1.3)
Taste of the low-salt food	171 (54.6)	96 (30.7)	40 (12.8)	5 (1.6)	1 (0.3)
Insufficient awareness of which foods to avoid	160 (51.1)	106 (33.9)	40 (12.8)	5 (1.6)	2 (0.6)
Lack of information regarding the risks of consuming a high-sodium diet	159 (50.8)	96 (30.7)	38 (12.1)	16 (5.1)	4 (1.3)

**Figure 1:**
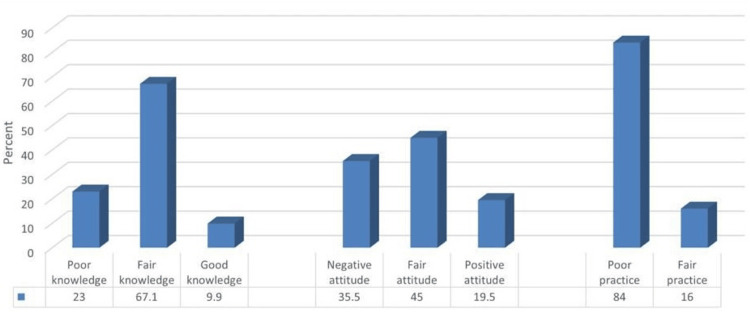
Percentage distribution of the participants according to their knowledge, attitude, and practice levels related to dietary salt intake (n=313). Data are presented as percentages (%).

A score of “one” was given to the right answer for knowledge, positive attitude, and correct practice. The scores of knowledge, attitude, and practice average were 16.99 ± 3.8, 3.03 ± 1.46, and 2.13 ± 1.34, respectively. The percentage of poor, fair, and good knowledge levels regarding dietary salt intake among studied students was 72:23%, 210:67.1%, and 31:9.9%. While the prevalence of negative, fair, and positive attitudes was 111:35.5%, 141:45%, and 161:9.5%. As for practice level, none of the students had good practice, while 263:84% and 50:16% had poor and fair practices, respectively, as shown in Figure 1.

It was also found that the fair and positive attitudes of the students were those who had an older mean age, and those who had a positive attitude were in the fourth academic year (p<0.05), as demonstrated in Table 7. On the other side, it was found that the relationship between participants’ knowledge or practice levels and their age, gender, or academic level was non-significant (p=0.05).

**Table 7:** Relationship between participants' knowledge, attitude, and practice levels and their age, gender, and academic level (n=313). *: Mann-Whitney test; **: Kruskal-Wallis test.

Variable				χ^2^	p-value
	Knowledge level				
	Poor no. (%)	Fair no. (%)	Good no. (%)		
Age	21.39 ± 1.83	21.47 ± 1.8	22.23 ± 2.81	2*	0.373
Gender				4.79	0.091
Female	34 (47.2)	125 (59.5)	21 (67.7)		
Male	38 (52.8)	85 (40.5)	10 (32.3)		
Academic year				11.88	0.156
2nd	16 (22.2)	40 (19)	3 (9.7)		
3rd	16 (22.2)	43 (20.5)	4 (12.9)		
4th	19 (26.4)	52 (24.8)	5 (16.1)		
5th	8 (11.1)	34 (16.2)	6 (19.4)		
6th	13 (18.1)	41 (19.5)	13 (41.9)		
	Attitude level				
	Negative no. (%)	Fair no. (%)	Positive no. (%)		
Age	21.18 ± 1.87	21.84 ± 1.97	21.43 ± 1.9	2*	0.03
Gender				5.44	0.066
Female	59 (53.2)	91 (62.5)	30 (49.2)		
Male	52 (46.8)	50 (35.5)	31 (50.8)		
Academic year				18.73	0.016
2nd	31 (27.9)	15 (10.6)	13 (21.3)		
3rd	25 (22.5)	31 (22)	7 (11.5)		
4th	23 (20.7)	35 (24.8)	18 (29.5)		
5th	12 (10.8)	24 (17)	12 (19.7)		
6th	20 (18)	36 (25.5)	11 (18)		
	Practice level				
	Poor no. (%)	Fair no. (%)	Good no. (%)		
Age	21.47 ± 1.78	21.8 ± 2.63		0.11**	0.906
Gender				1.37	0.241
Female	155 (58.9)	25 (50)			
Male	108 (421.4)	25 (50)			
Academic year				1.87*	0.759
2nd	47 (17.9)	12 (24)			
3rd	54 (20.5)	9 (18)			
4th	63 (24)	13 (26)			
5th	40 (15.2)	8 (16)			
6th	59 (22.4)	8 (16)			

**Figure 2:**
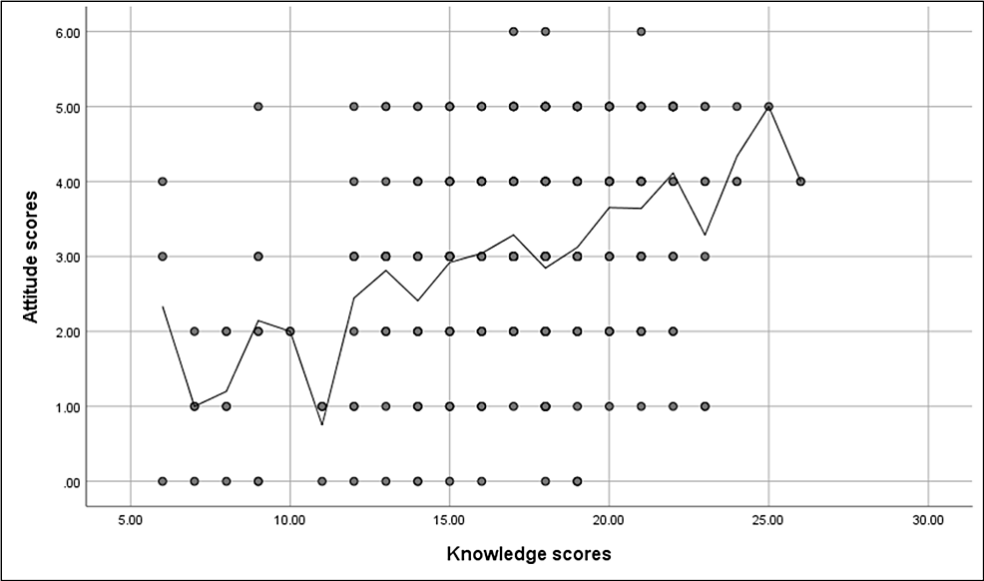
Spearman's correlation analysis between knowledge and attitude scores. r=0.35, p-value<0.001.

We found that knowledge and attitude have positive significant correlation scores (r=0.35, p-value<0.001), as illustrated in Figure 2. While the knowledge and practice scores show a non-significant correlation between attitude and practice, the scores were p=0.05.

## Discussion

This study sought to assess the knowledge, attitude, and practice toward dietary salt intake among medical students at KAU and identify barriers related to optimum dietary salt intake. According to the findings of our survey, participants had a moderate level of knowledge and understanding about dietary salt intake. In contrast, many previous studies found limited knowledge levels [12-14,17,18].

The vast majority of 300 (95.8%) of the students were also aware that serious health consequences can arise from consuming large amounts of salt, especially hypertension and kidney stones. This finding is compatible with past literature. In a study done in Lebanon targeting adult supermarket consumers, most of the study participants (93%) identified that a high-salt diet is an important risk factor for hypertension [12]. Another study done at a Malaysian university demonstrated that 98% of students were convinced that high salt consumption can have serious health consequences [17].

However, the majority of students did not know the recommendations of daily salt intake. In a cross-sectional study of medical professionals in Mongolia, it was found that older participants were considerably less likely to be unaware of the recommended dietary salt intake [18]. In this regard, our study did find a significant relationship between knowledge levels and students’ ages.

Regarding the attitude related to salt intake, the majority of students agreed that it is important to reduce salt intake, which is similar to the findings of the University of Sharjah [13]. This could be due to the fair knowledge they have about serious health problems caused by high salt intake. The findings of our study also revealed that fewer than a quarter of the students would check food labels for salt levels. These results align with those mentioned in the Sharjah University study [13] and are significantly less than those reported in Australia and Lebanon [11,12].

This suggests that students did not find salt to pose a significant health risk; this point is further supported by the fact that only 58 students (18.5%) reported that they would often attempt to lower the level of salt in their food, which represents a low percentage when compared to the approximately 96% of those who thought salt reduction was important. Alternatively, these results could be correlated with the fact that the majority of students found food labels to be unclear.

In addition, our findings demonstrated that the most common barrier to reducing salt consumption in the diet is the fact that salt enhances food ﬂavor, with more than half (171:54.6%) of the students agreeing on that. This was consistent with a study that was conducted on Saudi adults who sought primary health care in Mecca city [19], as half of the participants in that study stated that the main barrier to decreasing their salt intake was the taste of low-salt food and how salt makes food taste more delicious.

Limitations and recommendations

There are a few limitations of this study that must be recognized. The use of a self-reported questionnaire could have a recall bias and not accurately reflect beliefs and behaviors. In addition, the use of a cross-sectional study design could observe the association between variables but not the casual relationships. Also, the results only included medical students of King Abdulaziz University, which cannot be generalized to the broader population. Overall, the students' levels of awareness and behaviors for limiting dietary salt intake are fair. Cooperation between the health ministry and food producers may help Saudi Arabia's citizens consume less salt. This may be accomplished through raising public awareness and enacting legislation to set limits on the quantity of salt allowed in food products.

## Conclusions

In conclusion, the majority of students were aware that a high-salt diet can result in serious health issues. They were also uncertain of whether their salt intake was extremely high or not, which is consistent with the fact that they were ignorant of the daily salt intake guidelines. Moreover, additional care should be taken when generalizing the results since the study was conducted on medical students at King Abdulaziz University. The findings in our study can stand as a reference point for salt-related KAP studies to help further future researches in Saudi Arabia and other countries. We believe that multi-sector coordination between food suppliers, health agencies, and the government is necessary to increase public awareness and decrease the salt content of foods to lower individual salt consumption in Saudi Arabia.
